# Postural Body Sway as Surrogate Outcome for Myelopathy in Adrenoleukodystrophy

**DOI:** 10.3389/fphys.2020.00786

**Published:** 2020-07-17

**Authors:** Wouter J. C. van Ballegoij, Stephanie I. W. van de Stadt, Irene C. Huffnagel, Stephan Kemp, Marjo S. van der Knaap, Marc Engelen

**Affiliations:** ^1^Department of Pediatric Neurology, Amsterdam Leukodystrophy Center, Emma Children’s Hospital, Amsterdam University Medical Centers, University of Amsterdam, Amsterdam, Netherlands; ^2^Department of Neurology, OLVG Hospital, Amsterdam, Netherlands; ^3^Laboratory Genetic Metabolic Diseases, Department of Clinical Chemistry, Amsterdam University Medical Centers, Amsterdam Gastroenterology & Metabolism, University of Amsterdam, Amsterdam, Netherlands; ^4^Department of Functional Genomics, Vrije Universiteit Amsterdam, Amsterdam, Netherlands

**Keywords:** X-linked adrenoleukodystrophy, myelopathy, spinal cord, balance, body sway, surrogate outcome

## Abstract

**Background:**

Myelopathy is the core clinical manifestation of adrenoleukodystrophy (ALD), which is the most common peroxisomal disorder. Development of therapies requires sensitive and clinically relevant outcome measures. Together with spastic paraparesis, balance disturbance is the main cause of disability from myelopathy in ALD. In this cross-sectional study, we evaluated whether postural body sway – a measure of balance – could serve as a surrogate outcome in clinical trials.

**Methods:**

Forty-eight male ALD patients and 49 age-matched healthy male controls were included in this study. We compared sway amplitude and sway path of ALD patients to controls. We then correlated the body sway parameters showing the largest between-group differences with clinical measures of severity of myelopathy. To correct for age, we performed multiple linear regression analysis with age and severity of myelopathy as independent variables.

**Results:**

All body sway parameters were significantly higher in patients than in controls, with medium to large effect sizes (*r* = 0.43–0.66, *p* < 0.001). In the subgroup of asymptomatic patients, body sway amplitude was also higher, but the difference with controls was smaller than for symptomatic patients (effect size *r* = 0.38–0.46). We found moderate to strong correlations between body sway amplitude and clinical severity of myelopathy (*r* = 0.40–0.79, *p* < 0.005). After correction for age, severity of myelopathy was a significant predictor of body sway amplitude in all regression models.

**Conclusions:**

These results indicate that postural body sway may serve as a surrogate outcome for myelopathy in ALD. Such outcomes are important to evaluate new therapies in clinical trials. Further longitudinal studies are needed and ongoing in this cohort.

## Introduction

Progressive myelopathy affects almost all men with X-linked adrenoleukodystrophy (ALD) (Moser et al., 2001; Huffnagel et al., 2019b). ALD is a genetic neurometabolic disorder with an estimated incidence of 1 in 17000 (Bezman et al., 2001). It is caused by mutations in the ABCD1 gene that encodes the peroxisomal transmembrane transporter (referred to as ABCD1 protein) for very long-chain fatty acids (VLCFA) (Mosser et al., 1993; van Roermund et al., 2008). A defect in the ABCD1 protein results in impaired peroxisomal β-oxidation of VLCFA, leading to their accumulation in plasma and tissues, including the spinal cord (Igarashi et al., 1976; Powers et al., 2000). Symptoms of myelopathy typically start in the 3rd to 4th decades with a slowly progressive gait disorder (Engelen et al., 2012). Sphincter disturbance with both urinary and fecal incontinence is also frequently reported. On average, patients require a walking aid from the 6th decade and can eventually become wheelchair dependent (van Geel et al., 2001), making myelopathy the main cause of disability in ALD.

Development of disease-modifying therapies for myelopathy in ALD is hampered by a lack of reliable quantitative outcomes for clinical trials. Traditional clinical outcomes – such as the Expanded Disability Status Scale (EDSS), Severity Scoring system for Progressive Myelopathy (SSPROM), timed up-and-go test, and 6-minute walk test (6MWT) – are limited by their low sensitivity and high interrater and intrarater variability (Huffnagel et al., 2019b). Studies on more sophisticated surrogate outcomes such as magnetization transfer (MT) imaging (Fatemi et al., 2005), diffusion tensor imaging (DTI) (Huffnagel et al., 2019c), and optical coherence tomography (OCT) (van Ballegoij et al., 2020) provide evidence that they could be more sensitive and rater-independent. However, they lack direct clinical relevance, meaning that they are not of direct importance to the patient in terms of functional impairment or quality of life, while that is usually required for approval by regulatory agencies. Therefore, there is a need for surrogate outcomes that are both sensitive and clinically relevant.

The pathological hallmark of myelopathy in ALD is degeneration of the corticospinal tracts and dorsal columns of the spinal cord, causing spastic paraparesis and sensory ataxia (Powers et al., 2000). Sensory ataxia leads to an impaired balance, a key feature of the gait disorder in ALD (Moser et al., 2007). A measure of balance could, therefore, serve as a surrogate outcome in ALD. Indeed, Zackowski et al. (2006) showed that ALD patients with myelopathy have reduced balance compared to controls, as expressed by increased postural body sway amplitude measured with a force plate. This measurement of body sway is fast, non-invasive, and largely rater-independent, making it potentially suitable as surrogate outcome (Ruhe et al., 2010). It is also clinically relevant, as reduced balance directly contributes to disability in ALD. However, the number of patients in the Zackowski study was quite small (*n* = 20) and correlations with disease severity were not performed, leaving the value of body sway as surrogate outcome for myelopathy in ALD still largely undetermined.

In this cross-sectional study, we explored body sway as surrogate outcome for myelopathy in men with ALD. We compared body sway of ALD patients (symptomatic and asymptomatic) to a healthy age-matched control group. Moreover, we correlated body sway parameters with severity of myelopathy, measured by clinical and functional outcome measures.

## Materials and Methods

### Study Design and Participants

This single-center cross-sectional study was part of an ongoing observational cohort study on the natural history of ALD (the Dutch ALD cohort). For this particular study, patients were recruited at the Amsterdam UMC (Amsterdam, Netherlands) between January 2018 and December 2019. Male patients over 16 years of age with a confirmed diagnosis of ALD were eligible to participate. Exclusion criteria were inability to stand unsupported, active cerebral ALD (defined as gadolinium-enhancing cerebral white matter lesions on MRI), and any comorbidity interfering with the assessment of myelopathy, such as diabetes mellitus, neurodegenerative diseases (other than ALD), and a history of vertigo/vestibular disorder.

Study participation for patients included one hospital visit with neurological assessments, body sway measurement, and MR imaging. MRI scans to exclude active cerebral ALD were evaluated by an experienced neuroradiologist. Age-matched male controls without a history of diabetes or neurological or vestibular disease were recruited via public advertisement. All participants gave written informed consent prior to participation. The study protocol was approved by the local Institutional Review Board (METC 2014_302).

### Neurological Assessment

The protocol used to assess myelopathy in this cohort has been previously described (Huffnagel et al., 2019a, b). In short, patients underwent a detailed neurological history and examination. They were scored as symptomatic if they had both signs and symptoms of myelopathy; otherwise, they were scored as asymptomatic. We used clinical outcome measures to quantify myelopathy: the EDSS, Severity SSPROM, and 6MWT. The EDSS, originally designed to assess disability in multiple sclerosis but also widely used in ALD, measures neurological disability ranging from 0 (no disability) to 10 (death) (Kurtzke, 1983; Moser et al., 2004; Zackowski et al., 2006; Castellano et al., 2016; Huffnagel et al., 2019b). SSPROM measures severity of myelopathy ranging from 0 to 100, with lower scores indicating a higher degree of impairment (Castilhos et al., 2012; D’Souza et al., 2017). The 6MWT measures the maximum walking distance in 6 min and was performed on a 50-m flat indoor trail (van Hedel et al., 2005). Neurological assessments and body sway measurements were done on the same day.

### Measurement of Postural Body Sway

**FIGURE 1:**
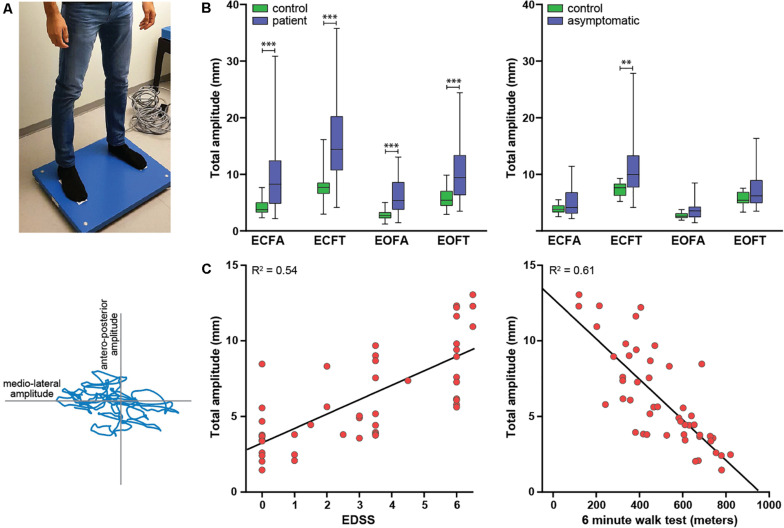
Study overview. **(A)** Experimental setup. Upper panel: subject standing on the force plate in the feet-apart condition. Lower panel: body sway output. The body sway amplitude is the displacement of the center of gravity in the anteroposterior (*y*-axis) or mediolateral (*x*-axis) direction, the sway path is the distance traveled by the blue line. **(B)** Differences in body sway amplitude between patients and controls (left) and asymptomatic patients and an age-matched selection of controls (right). **(C)** Two examples of the association between clinical severity of myelopathy and body sway: EDSS and total sway amplitude (left) and 6MWT and total sway amplitude (right). The lines represent simple linear regression lines.

Postural body sway was measured in the outpatient clinic by three operators using a Kistler force plate type 9260AA (Kistler Instrument AG, Winterthur, Switzerland) paired with Kistler’s Measurement, Analysis and Reporting software (MARS). The force plate dimensions were 60 × 60 × 5 cm, and the sampling frequency was 1000 Hz. The protocol consisted of two series of measurements in four conditions with a fixed sequence: eyes closed–feet apart, eyes open–feet apart, eyes closed–feet together, eyes open–feet together. Each measurement lasted 20 s; the mean of the two recordings per condition was used for the analysis. Recordings were performed in an adequately lit, quiet room with a hard and flat floor. We instructed subjects to take off their shoes and stand upright with their hands passively hanging. They were standing with their feet on visual markers at approximately shoulder width (feet-apart condition) or parallel immediately adjacent to each other (feet-closed condition). In the eyes-open condition, they were asked to keep focus on a visual marker placed on the wall approximately 2 m in front of them. During the recordings, subjects were to stand as still as possible and avoid any movements such as head movements, coughing, and talking. If the subject was not able to remain standing on the force plate in one of the conditions, this was recorded and the measurement in this condition was stopped. We used sway amplitude (total, anteroposterior, and mediolateral) and sway path (total, anteroposterior, and mediolateral) as parameters of postural sway (Figure 1A). Sway amplitude represents the average amount of the center of pressure (COP) sway in the anteroposterior or mediolateral direction and was calculated as the length of the trajectory of the COP sway in the anteroposterior or mediolateral direction divided by the number of changes in this direction (i.e., from moving forward to backward or vice versa). Sway path represents the length of the trajectory of COP over the support base divided by the measurement time (Baratto et al., 2002).

### Statistical Analysis

We used IBM SPSS statistics version 25 (IBM Inc.) for all statistical analyses. Normality was assessed with visual inspection of QQ plots and using the Shapiro–Wilk test (Shapiro and Wilk, 1965). Normally distributed data were presented as mean with standard deviation (SD); non-normally distributed data as median with interquartile range (IQR).

First, we assessed differences in body sway parameters between patients and controls with the Mann–Whitney *U*-test (non-normally distributed data). Second, we assessed differences in body sway parameters between asymptomatic patients and controls. As the prevalence of myelopathy in ALD increases with age, asymptomatic patients were significantly younger than the control group. Correction for the possible confounding effect of age through ANCOVA was not possible because the residuals of the asymptomatic group were not normally distributed. Therefore, we selected an equally sized sample of subjects from the control group, matched for age with the asymptomatic group. Subsequently, we compared body sway parameters between these groups using the unpaired t-test (normally distributed data) or Mann–Whitney *U*-test (non-normally distributed data). For both group comparisons, the effect size (*r*) was reported, which was calculated as the test statistic (*t*) divided by the square root of the number of patients for normally distributed data and as the test statistic (*z*) divided by square root of the number subjects for non-normally distributed data. An effect size < 0.3 was considered a small effect, 0.3–0.5 a medium effect, and >0.5 a large effect (Cohen, 1988; Lakens, 2013). Third, in patients, we correlated clinical outcome measures of severity of myelopathy with the body sway parameters that showed the largest between-group differences using Spearman’s rank-order correlation (non-normally distributed continuous data and ordinal data) with a Bonferroni correction for multiple comparisons. Finally, to control for a possible confounding effect of age, we performed multiple linear regression analyses with both age and clinical outcome measures of severity of myelopathy as independent variables and body sway parameters as dependent variables. Body sway parameters were not normally distributed, but the residuals were, thereby not violating the assumptions of linear regression analysis.

For all statistical tests, a significance level of α = 0.05 (two-sided) was chosen. Significance levels after correction for multiple comparisons were reported separately.

## Results

### Participant Characteristics

Of 103 subjects screened, 97 were included in the analysis: 48 patients and 49 healthy controls. Six patients were excluded: 3 because they were unable to stand unsupported (all were wheelchair-dependent), 2 because of active cerebral ALD, and 1 because of a technical problem during the measurement. None of the screened healthy controls were excluded. The mean age of the patient group was slightly higher than the control group (44.0 versus 41.4 years), but the difference was not statistically significant (*p* = 0.41), nor was there a difference in weight (79.1 versus 82.4 kg, *p* = 0.104). The healthy control group was significantly taller than the patient group (185 versus 180 cm, *p* = < 0.001).

Details on the neurological assessments in our cohort have been previously described (Huffnagel et al., 2019b). In short, for the patients included in this particular study, 32/48 (67%) were symptomatic, meaning that they had both signs and symptoms of myelopathy. The median score on the EDSS was 3.5 (IQR 0.25–6.0) and that on the SSPROM 87.3 (IQR 76.4–100), and the mean distance walked on 6MWT was 509.0 (SD 176.7) m.

### Body Sway Analysis

First, we assessed differences in body sway parameters between patients and controls. Six patients were not able to remain standing on the force plate in the eyes closed–feet together condition; therefore, 42 instead of 48 patients were included in this analysis. Patients had significantly higher sway amplitudes and longer sway paths in all four measured conditions (Table 1 and Figure 1B). For most parameters, effect sizes were large and slightly higher for sway amplitude than sway path.

**TABLE 1:** Differences in body sway parameters between patients and controls.

Eyes	Feet	Body sway parameter	Control (*n* = 49)	Patient (*n* = 48)	*p*-value	Effect size (*r*)
Closed	Apart	Amplitude – total	3.75(3.24−5.04)	8.25(4.81−12.45)	<0.001	0.56
		Amplitude – AP	2.99(1.89−4.56)	10.48(5.07−18.67)	<0.001	0.63
		Amplitude – ML	0.91(0.52−1.25)	2.26(1.34−5.33)	<0.001	0.58
		Path – total	258.0(192.0−328.1)	514.8(315.6−729.7)	<0.001	0.60
		Path – AP	217.4(160.2−262.9)	437.3(264.2−652.9)	<0.001	0.57
		Path – ML	102.6(75.7−129.6)	172.5(135.5−269.1)	<0.001	0.55
	Together^a^	Amplitude – total	7.70(6.57−8.52)	14.40(10.71−20.26)	<0.001	0.64
		Amplitude – AP	4.84(3.36−6.51)	11.86(7.63−18.79)	<0.001	0.60
		Amplitude – ML	6.41(4.84−9.26)	17.66(10.36−25.33)	<0.001	0.56
		Path – total	508.3(386.6−653.1)	957.4(611.0−1249.5)	<0.001	0.53
		Path – AP	290.1(221.5−390.9)	558.1(377.9−764.5)	<0.001	0.54
		Path – ML	329.7(260.2−432.8)	617.0(389.7−892.9)	<0.001	0.51
Open	Apart	Amplitude – total	2.71(2.26−3.32)	5.37(3.76−8.63)	<0.001	0.65
		Amplitude – AP	1.33(1.02−1.84)	2.70(2.07−4.37)	<0.001	0.66
		Amplitude – ML	0.56(0.37−0.75)	0.96(0.66−1.61)	<0.001	0.48
		Path – total	148.3(129.2−187.4)	213.0(189.5−307.3)	<0.001	0.58
		Path – AP	116.3(97.1−141.6)	164.7(153.0−238.6)	<0.001	0.60
		Path – ML	76.4(58.2−95.6)	100.9(80.7−190.5)	<0.001	0.43
	Together	Amplitude – total	5.43(4.43−7.07)	9.44(6.32−13.39)	<0.001	0.56
		Amplitude – AP	2.00(1.43−2.46)	4.71(2.81−6.58)	<0.001	0.58
		Amplitude – ML	2.69(1.91−4.17)	6.82(3.90−9.42)	<0.001	0.57
		Path – total	266.2(212.8−318.0)	434.3(336.2−585.4)	<0.001	0.57
		Path – AP	152.6(126.5−198.5)	261.7(171.7−350.5)	<0.001	0.53
		Path – ML	184.0(142.4−225.9)	290.8(218.4−357.3)	<0.001	0.56

*Body sway amplitude (mm) and body sway path (mm) values are summarized as median (interquartile range). Mann–Whitney U-test was used to compare groups. ^a^There were 42 measurements available for the patient group in the eyes closed–feet together condition, as 6 patients were not able to remain standing in this condition.*

Second, we compared body sway parameters between asymptomatic patients and an equally sized age-matched selection of healthy controls. The body sway parameters were higher in the asymptomatic patient group, but only the sway amplitudes in the eyes closed–feet together condition reached statistical significance (Table 2 and Figure 1B).

**TABLE 2:** Differences in body sway parameters between asymptomatic patients and controls.

Eyes	Feet	Body sway parameter	Control (*n* = 16)	Asymptomatic (*n* = 16)	*p*-value	Effect size (*r*)
Closed	Apart	Amplitude – total	3.81(3.29−4.53)	4.15(3.08−6.83)	0.353	0.18
		Amplitude – AP	2.53(1.84−3.77)	4.29(2.32−5.68)	0.061	0.33
		Amplitude – ML	0.99(0.75−1.26)	1.43(1.14−1.64)	0.056	0.34
		Path – total	233.0(198.9−376.4)	302.1(253.8−442.0)	0.128	0.27
		Path – AP	188.9(149.5−290.7)	242.7(166.5−367.9)	0.361	0.17
		Path – ML	122.7 (45.7)	145.0 (52.3)	0.210	0.23
	Together^*a*^	Amplitude – total	7.64(6.24−8.35)	9.95(7.70−13.34)	**0.008**	**0.46**
		Amplitude – AP	4.92 (2.41)	7.93 (4.73)	**0.030**	**0.40**
		Amplitude – ML	6.49 (2.63)	10.15 (6.24)	**0.043**	**0.38**
		Path – total	512.7(382.1−673.6)	606.8(462.5−863.0)	0.184	0.24
		Path – AP	319.0 (123.2)	440.9 (247.4)	0.088	0.31
		Path – ML	352.9(258.8−401.2)	386.3(308.3−577.7)	0.341	0.17
Open	Apart	Amplitude – total	2.60(2.29−3.12)	3.55(2.43−4.29)	0.110	0.29
		Amplitude – AP	1.39 (0.46)	1.79 (0.81)	0.096	0.30
		Amplitude – ML	0.64(0.51−0.75)	0.85(0.66−1.10)	0.080	0.31
		Path – total	161.9(141.2−212.7)	200.7(162.5−250.7)	0.184	0.24
		Path – AP	122.5(95.6−157.6)	145.2(118.2−172.9)	0.381	0.16
		Path – ML	87.9(76.6−108.0)	100.0(86.2−135.8)	0.239	0.21
	Together	Amplitude – total	5.43(4.91−6.87)	6.17(4.92−8.97)	0.171	0.25
		Amplitude – AP	2.09 (0.75)	2.74 (1.68)	0.178	0.25
		Amplitude – ML	2.78 (1.05)	3.86 (2.55)	0.133	0.28
		Path – total	284.1 (61.5)	329.6 (128.7)	0.215	0.23
		Path – AP	174.7 (54.3)	197.2 (86.8)	0.387	0.16
		Path – ML	185.1 (38.1)	217.1 (89.9)	0.204	0.23

*Body sway amplitude (mm) and body sway path (mm) values are summarized as median (interquartile range) or mean (standard deviation) depending on the distribution of data. Unpaired T-test (normally distributed data) or Mann-Whitney U-test (non-normally distributed data) was used to compare groups. Significant differences are displayed in bold text.*

Third, we correlated the body sway parameters that showed the largest between-group differences (i.e., sway amplitudes and not sway paths) with clinical outcome measures of severity of myelopathy. All of these parameters correlated moderately to strongly with severity of myelopathy (Spearman’s rho correlation coefficient > 0.6, *p* = < 0.001); correlations were strongest for the 6MWT compared to the other clinical outcome measures (Table 3 and Figure 1C).

**TABLE 3:** Correlations between severity of myelopathy and body sway amplitude in men with ALD.

Eyes	Feet	Body sway parameter		EDSS	SSPROM	6MWT
Closed	Apart	Amplitude – total	Spearman’s rho	0.71	−0.76	−0.69
			*p*-value	<0.001	<0.001	<0.001
		Amplitude – AP	Spearman’s rho	0.59	−0.56	−0.68
			*p*-value	<0.001	<0.001	<0.001
		Amplitude – ML	Spearman’s rho	0.77	−0.76	−0.80
			*p*-value	<0.001	<0.001	<0.001
	Together	Amplitude – total	Spearman’s rho	0.56	−0.56	−0.62
			*p*-value	<0.001	<0.001	<0.001
		Amplitude – AP	Spearman’s rho	0.75	−0.75	−0.74
			*p*-value	<0.001	<0.001	<0.001
		Amplitude – ML	Spearman’s rho	0.65	−0.59	−0.72
			*p*-value	<0.001	<0.001	<0.001
Open	Apart	Amplitude – total	Spearman’s rho	0.73	−0.72	−0.71
			*p*-value	<0.001	<0.001	<0.001
		Amplitude – AP	Spearman’s rho	0.74	−0.71	−0.76
			*p*-value	<0.001	<0.001	<0.001
		Amplitude – ML	Spearman’s rho	0.67	−0.70	−0.67
			*p*-value	<0.001	<0.001	<0.001
	Together	Amplitude – total	Spearman’s rho	0.62	−0.58	−0.67
			*p*-value	<0.001	<0.001	<0.001
		Amplitude – AP	Spearman’s rho	0.36	−0.40	−0.41
			*p*-value	<0.001	0.005	0.004
		Amplitude – ML	Spearman’s rho	0.62	−0.60	−0.66
			*p*-value	<0.001	<0.001	<0.001

*All correlations were calculated with Spearman’s rank order correlation test. After Bonferroni correction for multiple comparisons, correlations were considered significant if p < 0.025.*

**FIGURE 2:**
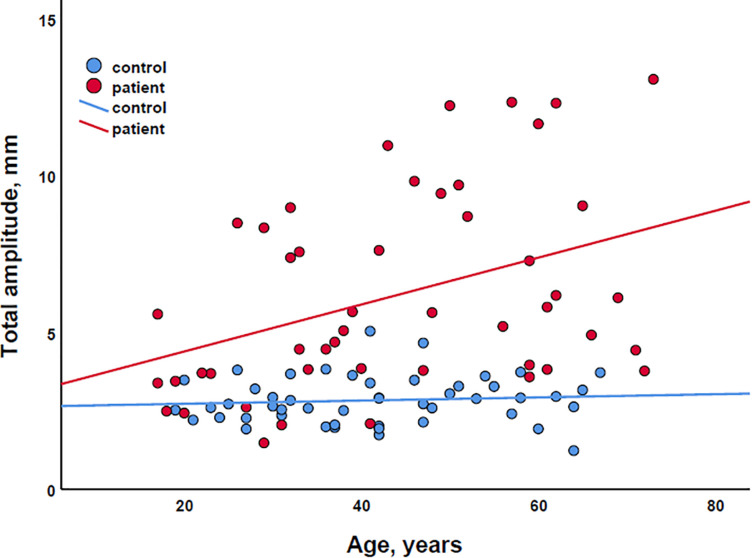
Scatter plot of the association between age and total sway amplitude for both patients (red) and controls (blue). The lines represent simple linear regression lines.

Finally, in exploratory scatter dot plots, we saw that there was an increase in most body sway parameters with age in both the patient and control groups (Figure 2). Therefore, to be able to correct for age, we performed multiple linear regression analysis with body sway amplitudes as dependent variables and (1) age and EDSS, (2) age and SSPROM, and (3) age and 6MWT as predictors. As expected, age and clinical parameters of severity of myelopathy were correlated (correlation coefficient between 0.59 and 0.68), but the correlation was below the regularly used cutoff value for collinearity (correlation coefficient > 0.8) – an important assumption for regression analysis (Vatcheva et al., 2016). In all three models, the clinical parameters of severity of myelopathy (EDSS, SSPROM, and 6MWT) were significant predictors of body sway amplitude (Supplementary Material). Conversely, age was a significant predictor for only three parameters: total and mediolateral sway amplitude in the model with EDSS and eyes closed–feet together condition, and total sway amplitude in the model with SSPROM and eyes closed–feet apart condition.

## Discussion

In this cross-sectional study, we explore the potential of postural body sway as surrogate outcome for myelopathy in ALD. We provide evidence that male ALD patients have significantly higher postural body sway than healthy controls and that body sway is also increased in clinically asymptomatic patients. Moreover, body sway parameters correlated strongly with clinical measures of severity of myelopathy.

Our results are in line with the study of Zackowski et al. (2006), who demonstrated an increased body sway amplitude in 20 ALD patients compared to healthy controls. Apart from this study, no studies on body sway as a measure of myelopathy in ALD are available. In hereditary spastic paraplegia, a myelopathy resembling that in ALD, postural body sway was significantly higher than in healthy controls and was correlated with muscle strength in the legs (Marsden and Stevenson, 2013). In cervical spondylotic myelopathy, the most common myelopathy, postural body sway was also increased (Yoshikawa et al., 2008; Haddas et al., 2019b) and improved after decompressive surgery (Haddas et al., 2019a). Although these conditions do not have the same pathophysiology as ALD, they indicate that postural sway could be a useful way to measure myelopathy.

As the balance disturbance in ALD is primarily caused by degeneration of the dorsal columns of the spinal cord that relay the proprioceptive information from the legs (Powers et al., 2000), one would expect it to be most pronounced in the “eyes closed” condition. In the “eyes open” condition, the patient can use his visual input to compensate for the lack of proprioceptive information. Similarly, a bigger difference with the control group could be expected in the more difficult “feet together”’ than the “feet apart” condition. However, although the absolute body sway values were indeed higher in both the “eyes closed” and “feet together” conditions, the differences between patients and controls were very similar for all four conditions (effect sizes around 0.5–0.6, Table 1), indicating that balance is severely affected in all conditions for the total patient group. By contrast, for the asymptomatic group, only the “eyes closed–feet together” condition showed significant between-group differences in sway amplitude (Table 2). Asymptomatic patients, although by definition not having any symptoms of myelopathy, frequently do have subtle signs of dorsal column dysfunction on neurological examination such as decreased vibration sense in the legs (Huffnagel et al., 2019b). This probably explains why their body sway amplitude is higher when tested in the most difficult condition. The fact that body sway is sensitive enough to detect changes in asymptomatic patients is important, because it could enable evaluation of disease-modifying therapies in the presymptomatic state – before any disability appears.

Although sensitive, postural body sway is not specific for myelopathy. For example, most male ALD patients also develop peripheral neuropathy (Kemp et al., 2016). The signs and symptoms of myelopathy are usually more severe, masking this neuropathy. However, the neuropathy does contribute to the balance disturbance. When measuring the effect of a disease-modifying therapy directed at the myelopathy (and not the peripheral neuropathy), one would not know if a change in postural body sway was caused by progression of the myelopathy or neuropathy. Similarly, body sway can be influenced by comorbidities such as cerebellar or vestibular disorders but also by motor or sensory deficits from for example cerebrovascular disorders. It is important to take such conditions into account and exclude subjects if necessary. Finally, application of body sway as surrogate outcome is also limited by disability, as it cannot be used for more severely affected and wheelchair-bound patients. This so-called ceiling effect is, however, also a problem for other outcome measures such as the 6MWT and DTI (Huffnagel et al., 2019c).

There are several potential sources of bias in our study. First, postural body sway is known to increase with age (Yoon et al., 2012). For the group comparisons, this should not be a problem as groups were matched for age. For the association with disease severity, we corrected for age through multiple linear regression analysis. However, age and disease severity were correlated, as both prevalence and severity of myelopathy in ALD increase with age. Although this correlation was below the commonly used threshold for collinearity in regression analysis (Vatcheva et al., 2016), correcting for age could have caused an underestimation of the association we found between body sway and disease severity. Second, height and weight can influence body sway, although studies show conflicting results (Hue et al., 2007; Yoon et al., 2012). Patients and controls in our cohort did not differ significantly in weight, but the healthy controls were significantly taller. Because we did not find an association between either height or weight and postural body sway in our cohort (data not shown), we decided not to correct for these parameters.

The strengths of our study are the fairly large sample size for such a rare disease, the use of an age- and sex-matched control group, and the comparisons with multiple, systematically collected clinical outcome measures. A limitation is that we did not evaluate test–retest reliability. In literature, however, it appears to be reasonable (Corriveau et al., 2001; Pinsault and Vuillerme, 2009) and – although beyond the scope of the current study – it may be included in our future studies.

In conclusion, in this study we provide evidence that myelopathy in ALD is associated with increased postural body sway, correlating strongly with disease severity. Body sway measurement is fast, non-invasive, largely rater-independent, and clinically relevant. It can be done in the outpatient clinic with automated analysis, enabling research in a multicenter setting, which is often needed in a rare disease like ALD. Therefore, postural body sway may serve as a new surrogate outcome for myelopathy in ALD. Further validation in a longitudinal design is needed and will be performed in this cohort.

## Data Availability Statement

The datasets generated for this study are available on request to the corresponding author.

## Ethics Statement

All participant gave written informed consent prior to participation. The studies involving human participants were reviewed and approved by the Medisch Ethische Toetsingscommissie AMC.

## Author Contributions

All authors made a substantial contribution to the work, contributed to the interpretation of the data, commented on previous versions of the manuscript, read, and approved the final manuscript. ME, WB, and IH performed the study design and conceptualization. WB and SS performed the data collection and analysis. WB wrote the first draft of the manuscript.

## Conflict of Interest

IH has received unrestricted research grants from Vertex. ME has received unrestricted research grants from Vertex, Swanbio, Bluebird Bio and Minoryx Therapeutics. SK has received unrestricted research grants from Swanbio and Bluebird Bio.

The remaining authors declare that the research was conducted in the absence of any commercial or financial relationships that could be construed as a potential conflict of interest.

## Abbreviations

6MWT: 6-minute walk test
ALD: adrenoleukodystrophy
AP: anteroposterior
DTI: diffusion tensor imaging
ECFA: eyes closed–feet apart
ECFT: eyes closed–feet together
EDSS: Expanded Disability Status Scale
EOFA: eyes open–feet apart
EOFT: eyes open–feet together
ML, mediolateral OCT: optical coherence tomography
SSPROM: Severity Scoring system for Progressive Myelopathy.

## References

[B1] Baratto L. Morasso P. G. Re C. Spada G. (2002). A new look at posturographic analysis in the clinical context: sway-density versus other parameterization techniques. *Mot. Contr.* 6 246–270. 10.1123/mcj.6.3.246 12122219

[B2] Bezman L. Moser A. B. Raymond G. V. Rinaldo P. Watkins P. A. Smith K. D. (2001). Adrenoleukodystrophy: incidence, new mutation rate, and results of extended family screening. *Ann. Neurol.* 49 512–517. 10.1002/ana.101 11310629

[B3] Castellano A. Papinutto N. Cadioli M. Brugnara G. Iadanza A. Scigliuolo G. (2016). Quantitative MRI of the spinal cord and brain in adrenomyeloneuropathy: in vivo assessment of structural changes. *Brain* 139(Pt 6), 1735–1746. 27068048 10.1093/brain/aww068

[B4] Castilhos R. M. Blank D. Netto C. B. Souza C. F. M. Fernandes L. N. T. Schwartz I. V. D. (2012). Severity score system for progressive myelopathy: development and validation of a new clinical scale. *Braz. J. Med. Biol.* 45 565–572. 10.1590/s0100-879x2012007500072 22570090 PMC3854272

[B5] Cohen J. (1988). *Statistical Power Analysis For The Behavioral Sciences.* Hillsdale, NJ: L. Erlbaum Associates.

[B6] Corriveau H. Hebert R. Prince F. Raiche M. (2001). Postural control in the elderly: an analysis of test-retest and interrater reliability of the COP-COM variable. *Archiv. Phys. Med. Rehabil.* 82 80–85. 10.1053/apmr.2001.18678 11239290

[B7] D’Souza M. Yaldizli O. John R. Vogt D. R. Papadopoulou A. Lucassen E. (2017). Neurostatus e-Scoring improves consistency of expanded disability status scale assessments: a proof of concept study. *Mult. Scler.* 23 597–603. 10.1177/1352458516657439 27364325

[B8] Engelen M. Kemp S. de Visser M. van Geel B. M. Wanders R. J. A. Aubourg P. (2012). X-linked adrenoleukodystrophy (X-ALD): clinical presentation and guidelines for diagnosis, follow-up and management. *Orphanet. J. Rare Dis.* 7:51. 10.1186/1750-1172-7-51 22889154 PMC3503704

[B9] Fatemi A. Smith S. A. Dubey P. Zackowski K. M. Bastian A. J. van Zijl P. C. (2005). Magnetization transfer MRI demonstrates spinal cord abnormalities in adrenomyeloneuropathy. *Neurology* 64 1739–1745. 10.1212/01.wnl.0000164458.02141.06 15911801

[B10] Haddas R. Ju K. L. Boah A. Kosztowski T. Derman P. B. (2019a). The effect of surgical decompression on functional balance testing in patients with cervical spondylotic myelopathy. *Clin. Spine Surg.* 32 369–376. 10.1097/bsd.0000000000000889 31498275

[B11] Haddas R. Lieberman I. Boah A. Arakal R. Belanger T. Ju K. L. (2019b). Functional balance testing in cervical spondylotic myelopathy patients. *Spine* 44 103–109. 10.1097/brs.0000000000002768 29958201

[B12] Hue O. Simoneau M. Marcotte J. Berrigan F. Doré J. Marceau P. (2007). Body weight is a strong predictor of postural stability. *Gait Post.* 26 32–38. 10.1016/j.gaitpost.2006.07.005 16931018

[B13] Huffnagel I. C. Dijkgraaf M. G. W. Janssens G. E. van Weeghel M. van Geel B. M. Poll-The B. T. (2019a). Disease progression in women with X-linked adrenoleukodystrophy is slow. *Orphanet J. Rare Dis.* 14:30. 10.1186/s13023-019-1008-6 PMC6367840 30732635

[B14] Huffnagel I. C. van Ballegoij W. J. C. van Geel B. M. Vos J. Kemp S. Engelen M. (2019b). Progression of myelopathy in males with adrenoleukodystrophy: towards clinical trial readiness. *Brain* 142 334–343. 10.1093/brain/awy299 30535170

[B15] Huffnagel I. C. van Ballegoij W. J. C. Vos J. Kemp S. Caan M. W. A. Engelen M. (2019c). Longitudinal diffusion MRI as surrogate outcome measure for myelopathy in adrenoleukodystrophy. *Neurology* 93:e02133-43. 10.1212/WNL.0000000000008572 31719133

[B16] Igarashi M. Schaumburg H. H. Powers J. Kishmoto Y. Kolodny E. Suzuki K. (1976). Fatty acid abnormality in adrenoleukodystrophy. *J. Neurochem.* 26 851–860. 10.1111/j.1471-4159.1976.tb04461.x-i1 965973

[B17] Kemp S. Huffnagel I. C. Linthorst G. E. Wanders R. J. Engelen M. (2016). Adrenoleukodystrophy - neuroendocrine pathogenesis and redefinition of natural history. *Nat. Rev. Endocrinol.* 12 606–615. 10.1038/nrendo.2016.90 27312864

[B18] Kurtzke J. F. (1983). Rating neurologic impairment in multiple sclerosis: an expanded disability status scale (EDSS). *Neurology* 33 1444–1452. 6685237 10.1212/wnl.33.11.1444

[B19] Lakens D. (2013). Calculating and reporting effect sizes to facilitate cumulative science: a practical primer for t-tests and ANOVAs. *Front. Psychol.* 4:863. 10.3389/fpsyg.2013.00863 24324449 PMC3840331

[B20] Marsden J. Stevenson V. (2013). Balance dysfunction in hereditary and spontaneous spastic paraparesis. *Gait Post.* 38 1048–1050. 10.1016/j.gaitpost.2013.03.001 23587557

[B21] Moser H. W. Fatemi A. Zackowski K. Smith S. Golay X. Muenz L. (2004). Evaluation of therapy of X-linked adrenoleukodystrophy. *Neurochem. Res.* 29 1003–1016. 15139299 10.1023/b:nere.0000021245.12181.90

[B22] Moser H. W. Mahmood A. Raymond G. V. (2007). X-linked adrenoleukodystrophy. *Nat. Clin. Pract. Neurol.* 3 140–151. 17342190 10.1038/ncpneuro0421

[B23] Moser H. W. Smith K. D. Watkins P. A. Powers J. Moser A. B. (2001). *The Metabolic and Molecular Bases of Inherited Disease.* New York, NY: McGraw-Hill.

[B24] Mosser J. Douar A. M. Sarde C. O. Kioschis P. Feil R. Moser H. (1993). Putative X-linked adrenoleukodystrophy gene shares unexpected homology with ABC transporters. *Nature* 361 726–730. 10.1038/361726a0 8441467

[B25] Pinsault N. Vuillerme N. (2009). Test-retest reliability of centre of foot pressure measures to assess postural control during unperturbed stance. *Med. Eng. Phys.* 31 276–286. 10.1016/j.medengphy.2008.08.003 18835738

[B26] Powers J. M. DeCiero D. P. Ito M. Moser A. B. Moser H. W. (2000). Adrenomyeloneuropathy: a neuropathologic review featuring its noninflammatory myelopathy. *J. Neuropathol. Exper. Neurol.* 59 89–102. 10.1093/jnen/59.2.89 10749098

[B27] Ruhe A. Fejer R. Walker B. (2010). The test-retest reliability of centre of pressure measures in bipedal static task conditions–a systematic review of the literature. *Gait Post.* 32 436–445. 10.1016/j.gaitpost.2010.09.012 20947353

[B28] Shapiro S. S. Wilk M. B. (1965). An analysis of variance test for normality (Complete Samples). *Biometrika* 52 591–611. 10.1093/biomet/52.3-4.591

[B29] van Ballegoij W. J. C. Kuijpers S. C. Huffnagel I. C. Weinstein H. C. Poll-The B. T. Engelen M. (2020). Optical coherence tomography shows neuroretinal thinning in myelopathy of adrenoleukodystrophy. *J. Neurol.* 267 679–687. 10.1007/s00415-019-09627-z 31720823 PMC7035302

[B30] van Geel B. M. Bezman L. Loes D. J. Moser H. W. Raymond G. V. (2001). Evolution of phenotypes in adult male patients with X-linked adrenoleukodystrophy. *Ann. Neurol.* 49 186–194. 10.1002/1531-8249(20010201)49:2<186::aid-ana38>3.0.co;2-r 11220738

[B31] van Hedel H. J. Wirz M. Dietz V. (2005). Assessing walking ability in subjects with spinal cord injury: validity and reliability of 3 walking tests. *Archiv. Phys. Med. Rehabil.* 86 190–196. 10.1016/j.apmr.2004.02.010 15706542

[B32] van Roermund C. W. Visser W. F. Ijlst L. van Cruchten A. Boek M. Kulik W. (2008). The human peroxisomal ABC half transporter ALDP functions as a homodimer and accepts acyl-CoA esters. *FASEB J.* 22 4201–4208. 10.1096/fj.08-110866 18757502

[B33] Vatcheva K. P. Lee M. McCormick J. B. Rahbar M. H. (2016). Multicollinearity in regression analyses conducted in epidemiologic studies. *Epidemiology* 6:227. 10.4172/2161-1165.1000227 PMC4888898 27274911

[B34] Yoon J. J. Yoon T. S. Shin B. M. Na E. H. (2012). Factors affecting test results and standardized method in quiet standing balance evaluation. *Ann. Rehabil. Med.* 36 112–118. 22506243 10.5535/arm.2012.36.1.112 PMC3309333

[B35] Yoshikawa M. Doita M. Okamoto K. Manabe M. Sha N. Kurosaka M. (2008). Impaired postural stability in patients with cervical myelopathy: evaluation by computerized static stabilometry. *Spine* 33 E460–E464. 18552660 10.1097/BRS.0b013e318178e666

[B36] Zackowski K. M. Dubey P. Raymond G. V. Mori S. Bastian A. J. Moser H. W. (2006). Sensorimotor function and axonal integrity in adrenomyeloneuropathy. *Arch. Neurol.* 63 74–80. 16401738 10.1001/archneur.63.1.74

