# Supplementary material for: Postural Body Sway as Surrogate Outcome for Myelopathy in Adrenoleukodystrophy
Source: Front Physiol. 2020 Jul 17;11:786. doi: 10.3389/fphys.2020.00786 (PMC7379508; doi:10.3389/fphys.2020.00786)
Supplement: Supplementary file 1 [file Table_1.DOCX]

|  |  |  | **Model 1** | | | **Model 2** | | | **Model 3** | |
| --- | --- | --- | --- | --- | --- | --- | --- | --- | --- | --- |
| **Eyes** | **Feet** | **Parameter** | **EDSS** | **Age** | **SSPROM** | | **Age** | **6MWT** | | **Age** |
| Closed | Apart | Amplitude - total | B=2.312, p=<0.0005 | B=-0.142, p=0.009 | B=-0.503, p=<0.0005 | | B=-0.125, p=0.008 | B=-0.026, p=<0.0005 | | B=-0.083, p=0.103 |
|  |  | Amplitude - AP | B=3.448, p=<0.0005 | B=-0.137, p=0.127 | B=-0.691, p=<0.0005 | | B=-0.086, p=0.305 | B=-0.040, p=<0.0005 | | B=-0.060, p=0.464 |
|  |  | Amplitude - ML | B=1.814, p=<0.0005 | B=-0.125, p=0.035 | B=-0.364, p=<0.0005 | | B=-0.099, p=0.076 | B=-0.023, p=<0.0005 | | B=-0.094, p=0.075 |
|  |  |  |  |  |  | |  |  | |  |
|  | Together | Amplitude - total | B=2.266, p=<0.0005 | B=-0.116, p=0.168 | B=-0.405, p=0.002 | | B=-0.067, p=0.423 | B=-0.030, p=<0.0005 | | B=-0.084, p=0.238 |
|  |  | Amplitude - AP | B=2.205, p=<0.0005 | B=-0.039, p=0.574 | B=-0.317, p=0.006 | | B=0.025, p=0.734 | B=-0.027, p=<0.0005 | | B=-0.013, p=0.819 |
|  |  | Amplitude - ML | B=3.793, p=<0.0005 | B=-0.075, p=0.598 | B=-0.584, p=0.012 | | B=-0.050 p=0.734 | B=-0.043, p=0.001 | | B=0.028, p=0.828 |
|  |  |  |  |  |  | |  |  | |  |
| Open | Apart | Amplitude - total | B=2.266, p=<0.0005 | B=-0.035, p=0.177 | B=-0.211, p=<0.0005 | | B=-0.014, p=0.598 | B=-0.014, p=<0.0005 | | B=-0.019, p=0.393 |
|  |  | Amplitude - AP | B=0.848, p=0.003 | B=-0.012, p=0.767 | B=-0.148, p=0.013 | | B=0.034, p=0.389 | B=-0.012, p=<0.0005 | | B=0.016, p=0.649 |
|  |  | Amplitude - ML | B=0.529, p=0.003 | B=-0.030, p=0.230 | B=-0.085, p=0.020 | | B=-0.013, p=0.576 | B=-0.008, p=<0.0005 | | B=-0.028, p=0.195 |
|  |  |  |  |  |  | |  |  | |  |
|  | Together | Amplitude - total | B=1.311, p=<0.0005 | B=-0.065, p=0.180 | B=-0.223, p=0.002 | | B=-0.029, p=0.542 | B=-0.017, p=<0.0005 | | B=-0.047, p=0.268 |
|  |  | Amplitude - AP | B=1.574, p=<0.0005 | B=-0.064, p=0.162 | B=-0.267, p=<0.0005 | | B=-0.020, p=0.655 | B=-0.019, p=<0.0005 | | B=-0.032, p=0.432 |
|  |  | Amplitude - ML | B=0.935, p=0.004 | B=0.015, p=0.744 | B=-0.167, p=0.012 | | B=0.036, p=0.404 | B=-0.013, p=<0.0005 | | B=0.019, p=0.625 |

**Supplementary Table 1.** Regression coefficients for multiple linear regression analysis with corresponding p-values. Three models were constructed: a model with EDSS and age (model 1), with SSPROM and age (model 2), and 6MWT and age (model 3) as independent variables; body sway amplitudes (mm) were the independent variables for all models.
